# Central nervous system posttransplant lymphoproliferative disorder following allogeneic hematopoietic stem cell transplantation successfully treated with combination therapy of acalabrutinib and immunochemotherapy: A case report and literature review

**DOI:** 10.1002/jha2.1078

**Published:** 2024-12-28

**Authors:** Peihao Zheng, Teng Xu, Xiaona Zuo, Xiaoyan Ke, Kai Hu

**Affiliations:** ^1^ Department of Lymphoma and Myeloma Research Center Beijing Gobroad Boren Hospital Beijing China; ^2^ GoBroad Healthcare Group Beijing Gobroad Boren Hospital Beijing China; ^3^ Pathology Department Beijing Gobroad Boren Hospital Beijing China

**Keywords:** Acalabrutinib, CNS, EBV, HSCT, PTLD

## Abstract

Here, we report a case of Epstein‐Barr virus‐positive central nervous system‐post‐transplant lymphoproliferative disorder (CNS‐PTLD) patient who failed to achieve complete metabolic remission (CMR) after successively trying a methotrexate‐based regimen combined with orelabrutinib or whole‐brain radiotherapy and encountered intracranial hemorrhage during orelabrutinib treatment. Ultimately, the patient achieved CMR after one cycle of acalabrutinib in combination with temozolomide, teniposide, liposomal doxorubicin, dexamethasone, and rituximab (TEDDi‐R). Following another cycle of TEDDi‐R treatment, he has been receiving acalabrutinib maintenance up to now and remained in CMR. The case may provide an effective treatment option for CNS‐PTLD patients in clinical practice.

## 1 INTRODUCTION

Post‐transplant lymphoproliferative disorder (PTLD) involving the central nervous system (CNS) is relatively rare, mostly characterized by aggressive B‐cell lymphoma, and is often closely associated with Epstein‐Barr virus (EBV) [1, 2, 3]. EBV+ CNS PTLD has a worse prognosis than EBV‐negative patients with median overall survival of 10.8 and 43 months, separately [3], and there is currently no standard treatment. Here, we present the diagnosis and treatment process of a patient diagnosed with CNS‐PTLD after allogeneic hematopoietic stem cell transplantation (HSCT) for acute myeloid leukemia (AML) and a review of the literature.

## 2 CASE PRESENTATION

**FIGURE 1:**
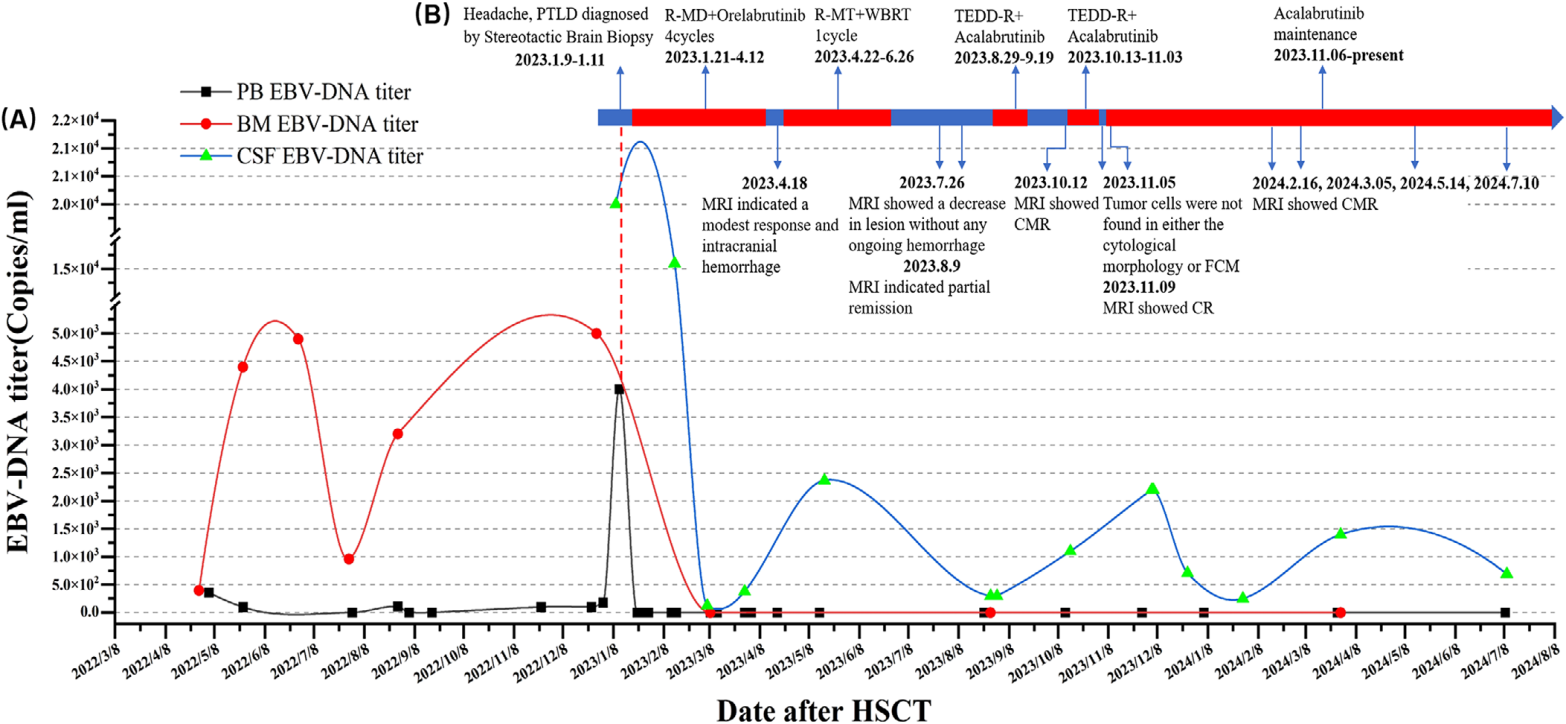
(A) The timeline of symptoms, imaging changes, and clinical interventions since diagnosis of post‐transplant lymphoproliferative disorder (PTLD). (B) The clinical treatment process of PTLD after hematopoietic stem cell transplantation (HSCT) and the changes of PB/BM/CSF EBV‐DNA titer.

A 32‐year‐old man was diagnosed with AML‐M2a on September 14, 2020. He underwent two cycles of induction chemotherapy with an IA regimen, achieving both morphologic and molecular remission. Then four cycles of consolidation therapy with medium dose cytarabine (MD‐Ara‐C) were initiated on January 7, 2021, comprising IA regimen (two cycles), HA regimen (1 cycle), and DA regimen (1 cycle) [4]. Morphological evaluation and flow cytometric (FCM) performed on February 11 indicated a complete morphological response, minimal residual disease (MRD) negative, quantification of NPM1 mutation at 0.084%, and CEBPA mutation negative. Then, he proceeded to haploidentical allogeneic HSCT from his daughter on March 8, 2022. Both leukocyte and platelet were engrafted on days +13 and +14 after HSCT, respectively. On day +51, bone marrow (BM) aspiration revealed morphological complete remission (CR), with negative MRD and complete chimerism. The BM EBV load was 4.0×10^2^ copies/mL, indicating an ongoing EBV infection within the BM, consequently necessitating the continuation of antiviral therapy. A subsequent BM aspiration reexamination nearly 3 months after HSCT showed the sustained remission of the underlying disease, with an EBV load of 4.4×10^3^ copies/mL, giving antiviral therapy and immunoglobulin transfusion. During this period, the BM consistently exhibited positivity for EBV (Figure 1A).

**FIGURE 2:**
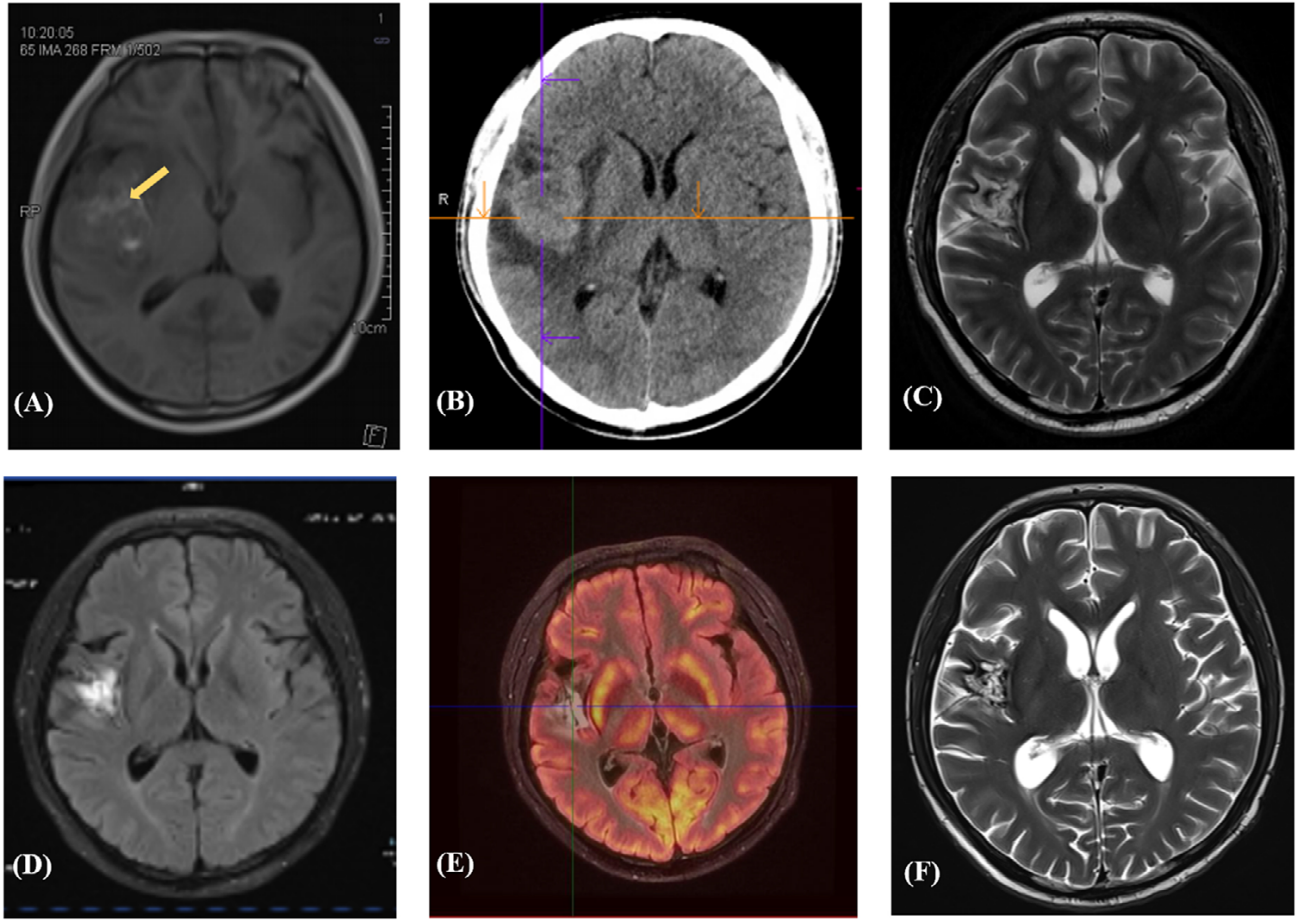
Brain images of central nervous system‐post‐transplant lymphoproliferative disorder (CNS‐PTLD). (A) Emergence of CNS‐PTLD. Positron emission tomography‐computed tomography (PET‐CT) revealed unevenly increased lesions in fluorodeoxyglucose (FDG) metabolism in the right temporal lobe (yellow arrow). The lesion measured was approximately 4.3 × 3.9 × 4.0 cm, SUVmax of 8.0, and central metabolism with SUVmax of 4.7. (B) Following R‐MD+orelabrutinib treatment for CNS‐PTLD. FDG metabolism in the right temporal lobe displayed irregularly heightened levels and a mixed‐density mass, consistent with lymphoma combined with hemorrhage. The intracranial lesions were smaller in size and slightly higher in metabolic activity than before: 1. the lesion measured was approximately 3.6 × 3.1 × 3.7 cm; 2. FDG uptake was unevenly increased with nodular‐like elevation, with SUVmax of 10.8 at the edge and slightly decreased central metabolism with SUVmax of 4.3. (C) Following immunochemotherapy +WBRT, nodular and patchy mixed signal shadows were observed in the right parietal lobe, indicating no further hemorrhage. (D) Three months after WBRT, abnormal signal shadows were visible in the right temporal lobe, with irregular morphology, measuring approximately 1.9×1.7×3.4 cm, which demonstrated partial remission. (E) After one cycle of TEDDi‐R treatment, abnormal signal shadows were observed in the right insular and temporal lobes, with no significant increase in FDG uptake, achieving CMR in the patient. (F) No significant enhancement was observed in the lesion by contrast‐enhanced MRI, which was similar to the MRIs on May 14, March 5, February 16, 2024, and November 10, 2023 (See Figure S1).

**FIGURE 3:**
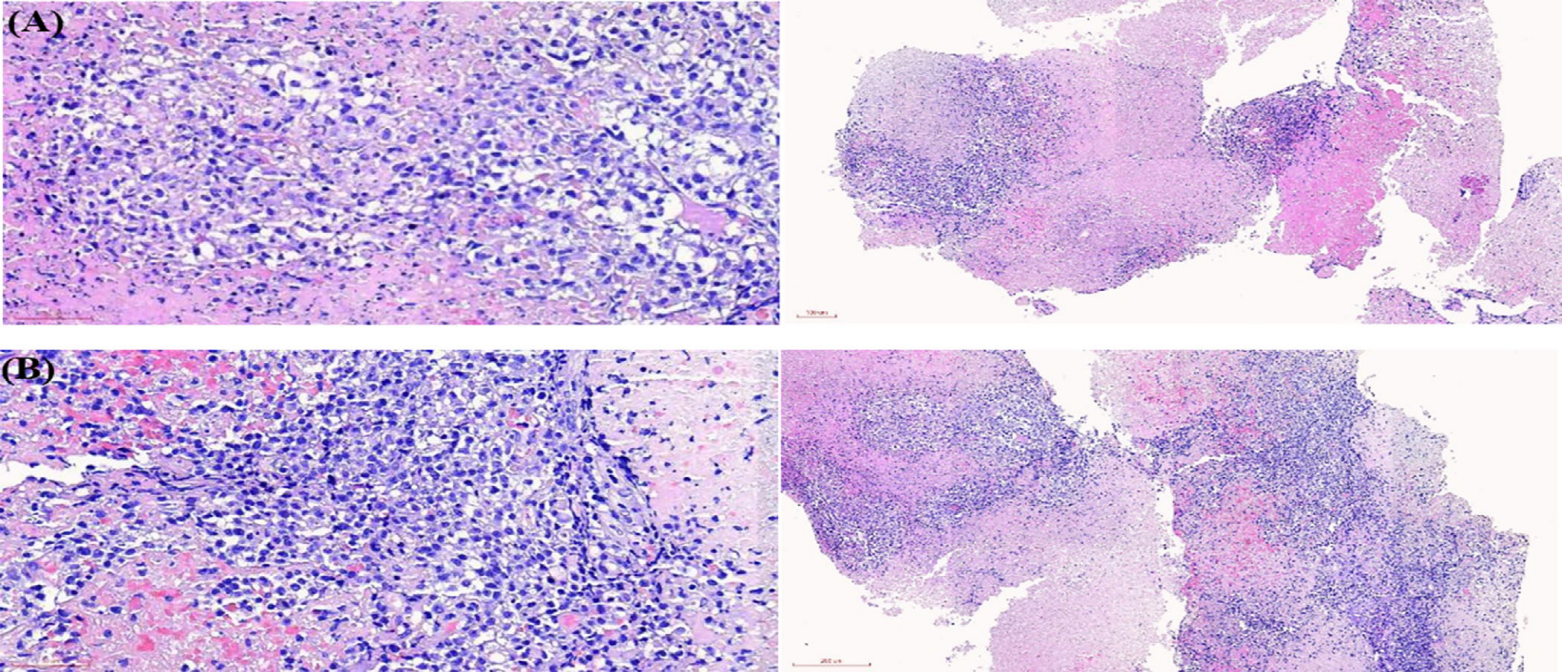
Pathological findings of stereotactic biopsy of the right temporal lobe lesion (January 11, 2023) (A) HE staining at 400x magnification. (B) HE staining at 100x magnification.

On January 9, 2023, the patient reported experiencing headaches and a physical examination showed no other neurological abnormalities. Positron emission tomography‐computed tomography (PET‐CT) in the emergency department showed a mixed high‐density lesion with surrounding edema in the right temporal lobe (Figure 2A). Subsequent lumbar puncture revealed negative FCM in the cerebrospinal fluid (CSF), with an EBV load of 2×10^4^ copies/ml. On January 11, a stereotactic brain lesion biopsy was performed, which showed a few medium‐sized atypical lymphoid cells with irregular karyotypes in a large area of necrotic tissue (intracranial mass in the right temporal lobe) (Figure 3). Immunohistochemistry analysis revealed the following profile: CD3(‐), CD20(+), MPO(‐), 01ig‐2(‐), GFAP(‐), Ki‐67(+50%); CD117(‐), CD99 (+), CD7(‐), Lysozyme(‐), CD68(‐), CD10(‐), Bc1‐6(‐), MUM‐1, Bc1‐2(+90%), c‐Myc(‐), EBER(+). FCM of brain tissue revealed 3.52% abnormal cells expressing CD19 and CD20 (among nucleated cells, *n* = 362), while lacking cKappa, cLambda, CD38, CD10, CD56, and CD5, which were suspected to be mature B cells with an abnormal phenotype. Based on the above examinations, the patient was diagnosed with CNS diffuse large B‐cell lymphoma with an IELSG score of 0 and a low MSKCC risk group.

**FIGURE 4:**
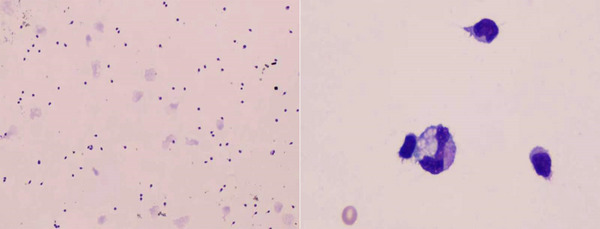
Cytological morphology of concentrated body fluid smear to detect tumor cells, which showed no tumor cells.

On January 21, R‐MD plus orelabrutinib was initiated, consisting of orelabrutinib at a daily dose of 150 mg for 21 days, methotrexate 2 g on day 1, rituximab 600 mg on day 1, and dexamethasone 20 mg on days 1–5. Subsequent positron emission tomography‐magnetic resonance imaging (PET‐MRI) on April 18 indicated the intracranial lesions were smaller in size and slightly higher in metabolic activity than before, indicative of persistent vigorous tumor activity. Furthermore, the fluorodeoxyglucose (FDG) metabolism in the right temporal lobe displayed irregularly heightened levels and a mixed‐density mass, consistent with lymphoma combined with hemorrhage, potentially linked to orelabrutinib (Figure 2B). Therefore, orelabrutinib was discontinued, and then the patient received one cycle of treatment with rituximab, high‐dose methotrexate, and temozolomide on April 22. From June 13 to 26, intensity‐modulated radiotherapy using 6MV X‐ray was performed at the outpatient department, the prescription dose was 30 Gy (1.5 Gy/time, two times a day, 5 days a week) to cover 95% of the planning clinical target volume (pCTV) and 40 Gy (2 Gy/time, two times a day, 5 days a week) targeting 95% of the planning gross target volume (pGTV) in 17 fractions. A follow‐up brain Contrast‐enhanced MRI (CE‐MRI) on July 26 showed a decrease in lesion size without any ongoing hemorrhage (Figure 2C). Subsequent CE‐MRI on August 9 indicated partial remission of the case (Figure 2D). On August 29, the patient received one cycle of TEDDi‐R regimen (acalabrutinib 100 mg twice a day, rituximab 500 mg on days 0–1, doxorubicin liposome 40 mg on day 1, dexamethasone 20 mg on days 1–5, teniposide 50 mg on days 1–4, temozolomide 200 mg on days 1–4). Concurrently, leucogen tablets were administered to mitigate the risk of bone marrow suppression. One week post‐chemotherapy, the patient experienced a significant decline in white blood cell and neutrophil counts, reaching a nadir of 1.7×10^9^ and 0.62×10^9^, respectively. In response, a granulocyte colony‐stimulating factor (G‐CSF) injection at a dosage of 150ug was administered for three consecutive days, leading to a subsequent increase in white blood cell and neutrophil counts to 4.1×10^9^ and 1.9×10^9^, respectively, while platelet counts remained within normal range. A PET‐MRI on October 12 confirmed a complete metabolic response (CMR) (Figure 1E). Following this, another cycle of the regimen was initiated on October 13th, with the patient's blood counts returning to normal prior to chemotherapy. Tumor cells were not detected in either cytological morphology or flow cytometry (FCM) (Figure 4). Subsequently, the patient has been on acalabrutinib maintenance up to the present, which has been well‐tolerated. The patient undergoes CE‐MRI and lumbar puncture to monitor EBV load in the cerebrospinal fluid approximately every 3 months. The latest MRI on July 9 continued to show CMR (Figure 1F). The patient's speech remains coherent, with no occurrence of headache or other abnormal neurological symptoms, and the Eastern Cooperative Oncology Group (ECOG) status remains at 0 points. As for CSV EBV load, it experienced a transient increase during immunochemotherapy but began to decrease during acalabrutinib maintenance (Figure 1A). The timeline of symptoms, imaging changes, and clinical interventions are summarized in Figure 1B.

## 3 DISCUSSION

In this case, the patient failed to achieve CMR after successively trying a methotrexate‐based regimen combined with orelabrutinib or whole‐brain radiotherapy, which may be a challenge for subsequent therapy. A previous study involving 18 CNS lymphoma patients (comprising five treatment‐naive individuals and 13 with relapsed or refractory disease) treated with TEDDi‐R regimen showed a high CR rate of 86% [5]. Based on these, the TEDDi‐R regimen was considered for the next treatment. Given the intracranial hemorrhage during previous orelabrutinib therapy, acalabrutinib may be a more suitable option, which is characterized by superior selectivity and lower risk of bleeding compared to ibrutinib and zanubrutinib [6, 7, 8]. As expected, the MRI showed CMR after one treatment cycle. Meanwhile, it was well tolerated and no bleeding event occurred.

The patient's peripheral blood EBV load remained negative since the initiation of CNS‐PTLD treatment in January 2023, but the CSF EBV remained intermittently positive. There was an increase observed during immunochemotherapy, it decreased to a lower level with continued acalabrutinib treatment (Figure 1A), which may be attributed to the killing effects of Bruton's tyrosine kinase inhibitor (BTKi) on EBV‐infected B cells [9, 10].

Previous research on EBV‐positive PTLD has found that B‐cell receptor (BCR) signaling is associated with the expansion of EBV by influencing the lytic viral's expression [10, 11]. The EBV membrane protein LMP2A mimics BCR signals, thereby sustaining EBV's latent infection within B cells. By activating a BCR‐like signaling pathway, LMP2A helps EBV‐infected B cells in evading apoptosis and facilitates viral persistence. BTK kinase occupies an important position in BCR signaling, and theoretically, due to the mimicry of BCR signaling by LMP2A, the combined use of BTKi may be effective in treating EBV‐associated B‐cell lymphoma [9]. In vitro experiments confirmed that ibrutinib can block BCR‐mediated EBV lytic viral expression, thereby impairing EBV‐infected B cells [10]. Thus, it's feasible to apply BTKi in treating EBV+ PTLD. Moreover, BTKi can penetrate the blood‐brain barrier, presenting a potential therapeutic prospect in CNS‐PTLD. Case reports illustrated the successful treatment of CNS‐PTLD with BTKi [12, 13]. Two patients with EBV‐positive CNS‐PTLD following kidney transplant (one refractory to methotrexate and the other unsuitable for methotrexate) were treated with ibrutinib for 12 months. They achieved remission within 2 months of initiating ibrutinib therapy and subsequently received consolidation treatment with third‐party EBV‐specific T cells between weeks 10 and 13. As of the last follow‐up, both patients have remained in remission for more than 34 months. Zanubrutinib led to PR with negative EBV loads in a patient with EBV‐positive CNS‐PTLD following allogeneic HSCT, which was not well treated with methotrexate + rituximab and WBRT. The duration of response is yet to be determined due to the short follow‐up. This was the first report to describe treatment of CNS‐PTLD with BTKi and immunochemotherapy followed by acalabrutinib maintenance, which sustained CMR for 10 months. However, it is not sure whether the response for PTLD is durable or further improved. Longer follow‐up is needed to evaluate its effect. Previous study indicates BTKi may have certain limitations in EBV+ PCNSL and there is no prognostic indicator analysis of BTKi for the patient, such as MCD genetic subtype or MYD88 and CD79B mutations [14]. But at this stage, the patient is still on acalabrutinib alone, and EBV‐specific T cells or some new therapies will be made after a continued increase in CNS EBV or disease progression.

## 4 CONCLUSION

Treatment for CNS‐PTLD typically involves high‐dose methotrexate‐based regimens, yet the overall prognosis remains unfavorable with limited scope for clinical trials. This case illustrates the TEDDi‐R regimen offers an effective and safe treatment option for patients with EBV‐positive CNS‐PTLD.

## CONFLICT OF INTEREST STATEMENT

The authors declare no conflict of interest.

## FUNDING INFORMATION

We have no funding to report for this article.

## ETHICS STATEMENT

The authors have confirmed ethical approval statement is not needed for this submission.

## PATIENT CONSENT STATEMENT

We obtained signed permission to publish a case report.

## CLINICAL TRIAL REGISTRATION

The authors have confirmed clinical trial registration is not needed for this submission.

## Supporting information

Supporting Information

## Data Availability

The original contributions presented in the study are included in the article. Further inquiries can be directed to the corresponding author.
