# Supplementary material for: Central nervous system posttransplant lymphoproliferative disorder following allogeneic hematopoietic stem cell transplantation successfully treated with combination therapy of acalabrutinib and immunochemotherapy: A case report and literature review
Source: EJHaem. 2024 Dec 28;6(1):e1078. doi: 10.1002/jha2.1078 (PMC11756970; doi:10.1002/jha2.1078)

**Central nervous system posttransplant lymphoproliferative disorder following allogeneic hematopoietic stem cell transplantation successfully treated with combination therapy of acalabrutinib and immunochemotherapy: a case report and literature review**

**Peihao Zheng^1^, Teng Xu^2^, Xiaona Zuo^3^, Xiaoyan Ke^1^, Kai Hu^1^***

^1^Department of Lymphoma and Myeloma Research Center, Beijing Gobroad Boren Hospital, Beijing, China

^2^GoBroad Healthcare Group, Beijing, China

^3^Pathology Department, Beijing Gobroad Boren Hospital, Beijing, China

**SUPPLEMENTARY MATERIAL**

Figure S1. Contrast-enhanced MRI on November 10, 2023 (A); February 16, 2024 (B); March 5, 2024 (C); May 14, 2024 (D). No significant enhancement was observed in the lesion.


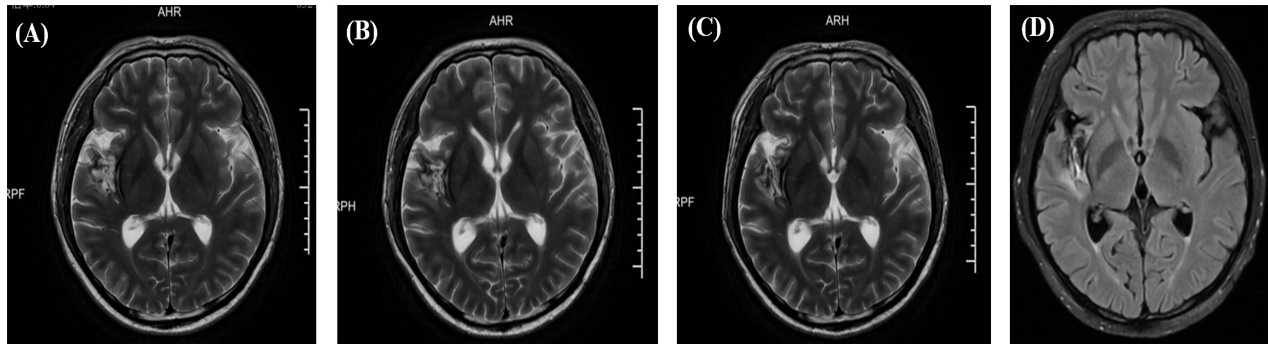

Supplement: Supplementary file 1 — Supporting Information [file JHA2-6-e1078-s001.docx]
